# A Brief Assessment of Conversation Skills in Adolescents and Adults with Autism Spectrum Disorder

**DOI:** 10.3390/bs16091492

**Published:** 2026-08-26

**Authors:** Faris R. Kronfli, Courtney Kenney, Timothy R. Vollmer, SungWoo Kahng

**Affiliations:** 1 Department of Psychology, University of Florida, Gainesville, FL 32611, USA; vollmera@ufl.edu; 2 Graduate School of Applied and Professional Psychology, Rutgers University, New Brunswick, NJ 08901, USA; courtney.kenney@rutgers.edu (C.K.); s.kahng@rutgers.edu (S.K.)

**Keywords:** assessment, autism spectrum disorder, conversations, social communication

## Abstract

Effective conversation skills are vital for navigating social interactions successfully. However, many individuals with autism spectrum disorder (ASD) face challenges in developing and maintaining these skills. We aimed to create a brief assessment to evaluate small talk among individuals with ASD. Three individuals diagnosed with ASD participated in the study. Assessment sessions were conducted in person, with questions asked at varying intervals to evoke participant responses. The assessment was effective in identifying potential conversation skills requiring improvement. The findings can inform the design of individualized and naturalistic interventions to enhance conversation skills in adolescents and adults with ASD, fostering improved social communication and engagement.

## 1. Introduction

Conversation skills are important for personal and professional development and may be considered a prerequisite to social acceptance, friendships, and employment opportunities. Given that social communication deficits are a core symptom of autism spectrum disorder (ASD; American Psychiatric Association, 2022), it is not surprising that engaging in appropriate conversations may be a difficult skill to develop or maintain for individuals with ASD. It is also one for which many individuals with ASD^1^ require assistance (Koegel et al., 2014).

Social and conversation skills are often taught using a manualized approach (Laugeson et al., 2015). For example, Laugeson et al.’s *PEERS for Young Adults Intervention* consisted of 16 weekly 90-min sessions. Although effective, it is possible that individuals interested in participating might not be able to commit to the program due to the duration. Although more brief, Custer et al. (2021) evaluated the use of a computer-based, 4-week training program to improve conversation skills in five adults with ASD. Results demonstrated improvements in almost all conversation skills, but participants were required to attend a 3 h meeting, two times per week across 4 weeks. Thus, commonly used social communication teaching strategies are either manualized and may not capture the individualized aspects of natural conversations or are strenuous on resources. In addition, interventions are not always preceded by systematic assessments that identify the specific conversation skills to improve; rather, the targets for treatment are often selected based on anecdotal reports or assumptions (Hood et al., 2021). As a result, meaningful and generalizable assessments and interventions for social communication need to be addressed.

The purpose of the current study was to develop an efficient assessment tool for use with adolescents and young adults diagnosed with ASD. Importantly, all participants had independently sought assistance with social communication skills. The current assessment aimed to identify existing repertoires of social communication and was designed to pinpoint areas potentially in need of improvement.

## 2. Method

### 2.1. Participants and Settings

Jeff, Mariam, and Rya were the first three participants who agreed to participate in the study. Jeff was an 18-year-old male, Mariam was a 15-year-old female, and Rya was a 13-year-old female. They were recruited through a local autism clinic at which they were receiving behavior-analytic services to focus on various goals such as conversational, employment, and independent living skills (e.g., personal hygiene, safety skills, money management). All participants had expressed interest in participating and described conversational skills as something they considered needing improvement. Sessions were conducted in clinic rooms that included a couch, table, chairs, and some decorative items (e.g., faux plant, framed pictures of landscapes) and an audio recorder. Each assessment was conducted during a single 1 h weekly appointment with the first author. Sessions (for the assessment pertinent to this study) terminated after 5 min and a natural break in the conversation. The 1 h weekly appointments included other activities beyond the scope of this study. Although it did not occur, the session would have been terminated if it had been requested by the participant.

### 2.2. General Procedures

Prior to beginning the assessment, we used www.randomnoungenerator.com to identify ten topics that would be used for all participants. These topics included poems, climate, lakes, malls, rice, birds, education, clothes, and baseball and were introduced using the same sequence for all three participants. Once all ten topics were discussed, the sequence restarted again with poems. The reason for choosing random topics was that the goal was to evaluate whether the participant displayed the repertoire to engage in small talk across topics, whether preferred or not.

To begin the session, the experimenter said, “We are going to talk about [topic].” The experimenter would then start the session by asking the participant a question related to the topic. These questions occurred on a variable-time (VT) schedule of 60 s such that a question was asked at the start of the session, and then approximately around minutes 1, 2, 3, and 4 of the session, for a total of 5 questions per session. Questions were asked during natural points in the conversation. If the participant was speaking when the experimenter should have asked a question, the experimenter waited until a natural break in the conversation occurred before asking the question. This occasionally resulted in fewer than 5 questions emitted by the experimenter if the participant was talkative.

The purpose in limiting the frequency of questions asked was to ensure that the participant’s vocal behavior was under stimulus control of all vocal behavior emitted by the experimenter rather than only the questions asked. In other words, the experimenter sought to ensure that the participant’s spoken responses were influenced by all the things the experimenter said, not just the questions.

**Figure 1:**
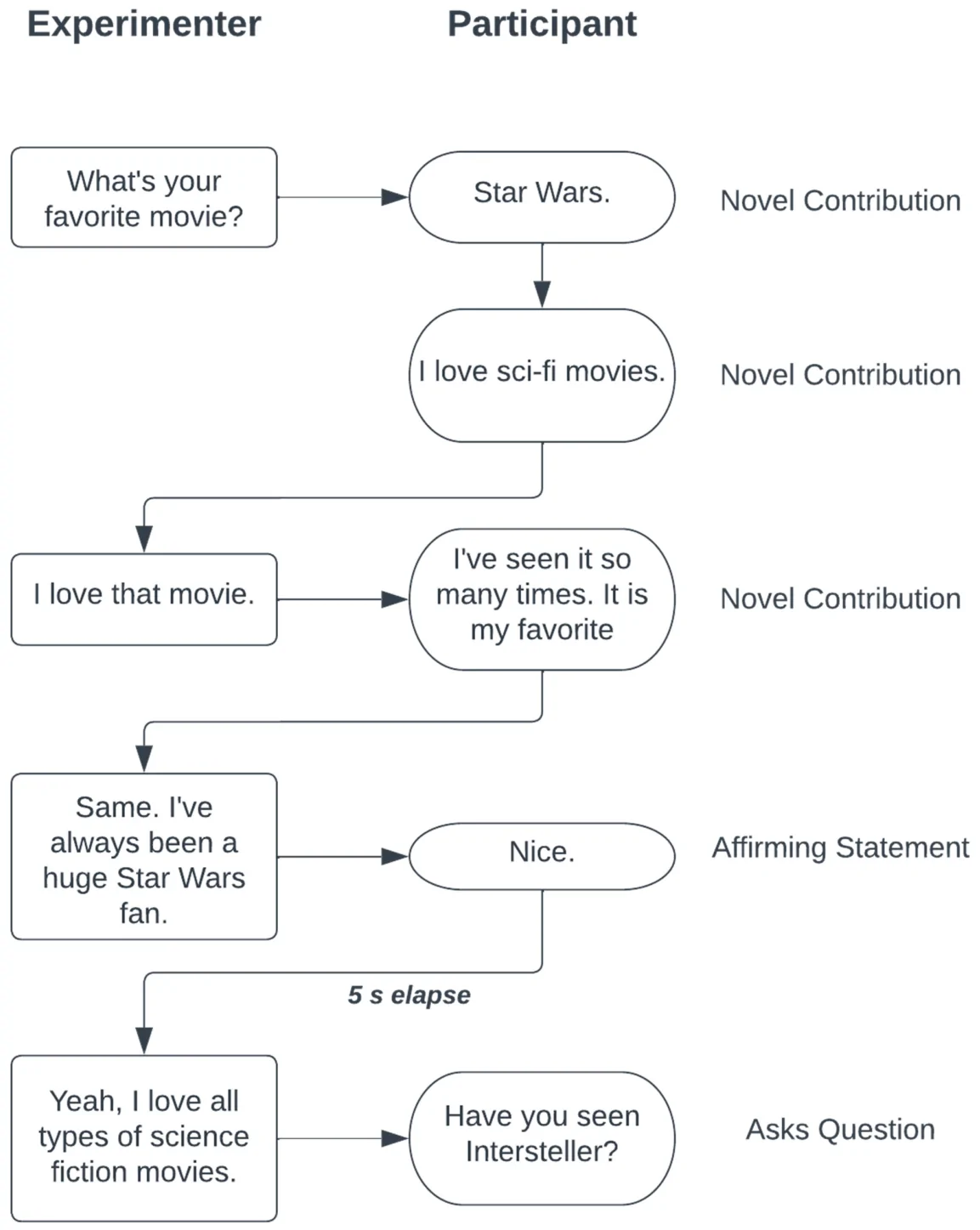
A flowchart depicting experimenter and participant behavior across a sample exchange in conversation.

The experimenter responded with positive affect and verbal affirmation across the session (e.g., “That is interesting!”) contingent on any vocalization the participant made related to the topic. If asked a question related to the topic, the experimenter answered it, also with positive affect (e.g., in response to “Do you play video games?” the experimenter might say, “No, but I enjoy watching people play!” or “Yes, my favorite game is _____”). If asked a question related to another topic, the experimenter responded as stated above, but redirected to the chosen topic (“Let’s get back to ______”). If 5 s elapsed without a participant vocalization, the experimenter made a pertinent comment with positive affect to support continuation of the interaction. For example, if the topic was baseball, the experimenter may say, “I used to go to baseball games with my family when I was young.” Similarly, if the topic was the mall, the experimenter may say, “I really only enjoy going to the mall when I have a lot of shopping to do during the holidays.” Figure 1 shows an additional example of a conversation exchange.

*Novel Vocalizations* were defined as statements that contributed information to the conversation, and they were scored using a 10 s partial-interval recording system. These included responses to questions. *Affirming Vocalizations* were defined as acknowledgment responses that did not contribute novel vocal behavior to the conversation and were scored using a 10 s partial-interval recording system. *Questions Asked* included mands for information by the participant and were scored using a frequency measure. *Experimenter Vocalizations* were collected for all participants to monitor consistency in conversational opportunities and were defined as any vocal utterance (e.g., questions asked or answered, novel or affirmative vocalizations) emitted by the experimenter. *Experimenter Vocalizations* are only depicted for Rya because caregiver reports specifically identified conversation-partner characteristics as a potential variable influencing her conversational responding. Similar concerns were not reported for Jeff or Mariam, nor were they observed during sessions; therefore, analysis of conversation-partner characteristics was not conducted for those participants.

It is important to note that it was possible to score multiple behaviors in the same interval. For example, if we scored novel vocalizations, we might also score affirmative vocalizations within the same interval. Furthermore, “fillers” such as “Uhh” or “Umm” were not coded as vocalizations for any topography.

Novel vocalizations and affirmative vocalizations were scored using a 10 s partial-interval recording system to efficiently sample conversational behavior while allowing simultaneous measurement of multiple responses occurring during interactions. Partial-interval recording also permitted the scoring of behavior that varied in duration and frequency across conversational exchanges without requiring exact measurement of vocal responses, which may be resource-intensive.

Interval-by-interval interobserver agreement was calculated for approximately 20% of sessions across participants and phases. This is much lower than what is commonly recommended but was a result of the lack of personnel available for data collection. The number of intervals with agreement was divided by the total number of intervals and multiplied by 100. Novel vocalizations averaged 93.3% (range, 83.3–100), affirmative vocalizations averaged 91.5% (range, 83.3–100), questions asked averaged 97.3% (range, 90–100), and experimenter vocalizations averaged 85.6% (range, 93.3–86.7).

### 2.3. Behavioral Skills Training

Although the primary purpose of the study was to develop an assessment that would efficiently identify current conversation skill repertoires, we did want to provide additional support for participants who were interested in working on social skills. Jeff and Rya expressed interest in progressing to behavioral skills training (BST) to better engage in conversations with a partner. BST was selected because it is an empirically supported approach for teaching conversation and related social-communication skills (Leaf et al., 2015). Participants completed a BST session to teach the importance of novel and affirmative vocalizations during a conversation in addition to asking questions. The training consisted of instruction, modeling, and rehearsal. During the instruction component, we described why these skills were important to reciprocate a conversation, regardless of the topic provided. We also explained that the purpose was not to require participants to speak about nonpreferred topics, but to have the skills necessary to reciprocate a conversation if they chose to do so. During the modeling component, we provided a live demonstration of the target behaviors for the participant to observe. We modeled asking and answering questions, affirming statements, and novel contributions. We also allowed participants the opportunity to ask questions regarding the modeled behavior observed. During the rehearsal component, the participant practiced the behaviors while receiving constructive and corrective feedback from the experimenter. BST required approximately 10 to 20 min to complete (see Kronfli et al., 2022). Jeff and Rya participated in multiple BST trials until meeting 100% mastery for two consecutive trials. Procedural fidelity of BST implementation was not formally assessed.

## 3. Results

**Figure 2:**
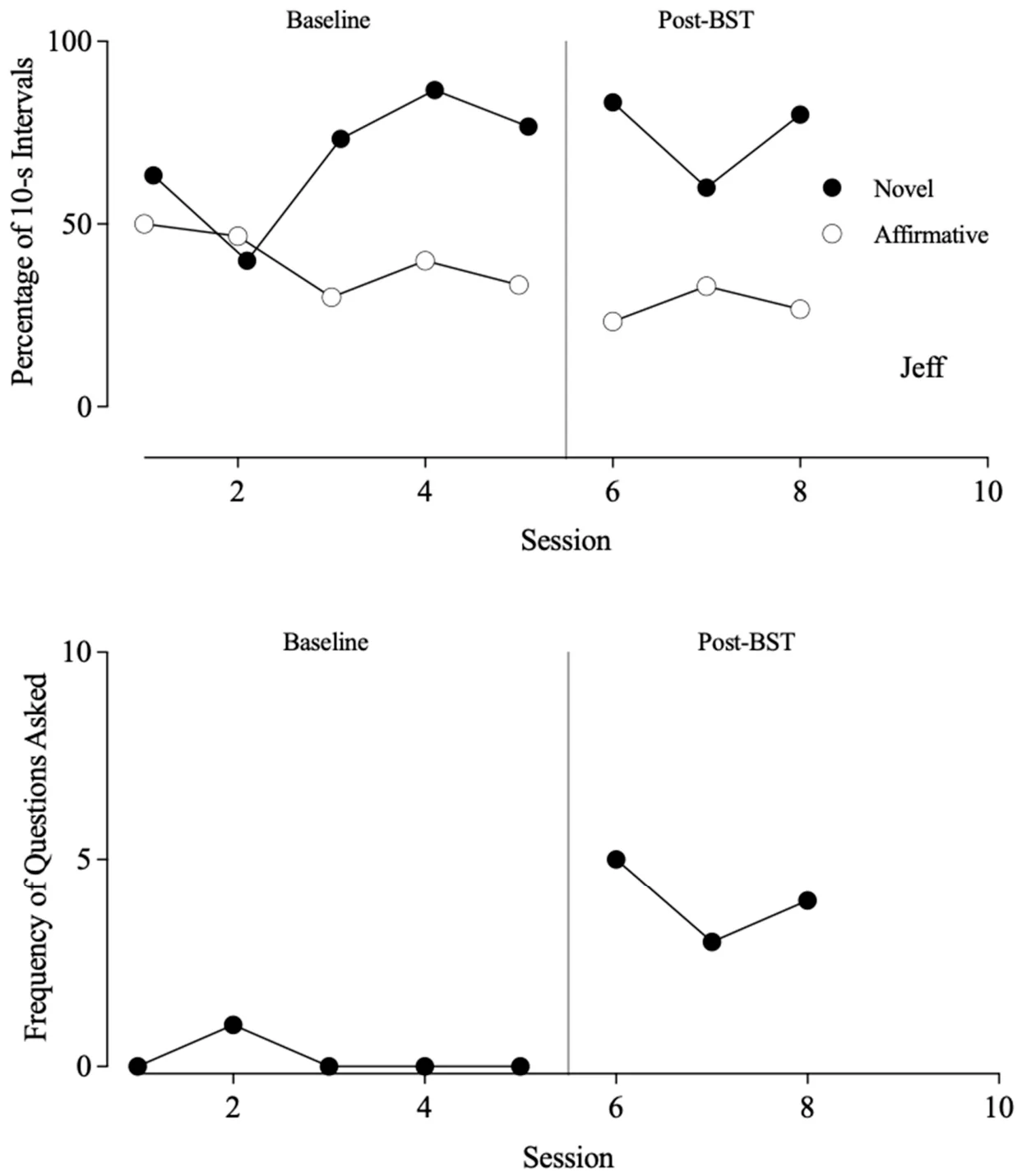
Percent of intervals with Novel and Affirming Vocalizations, and frequency of Questions Asked per session for Jeff.

**Figure 3:**
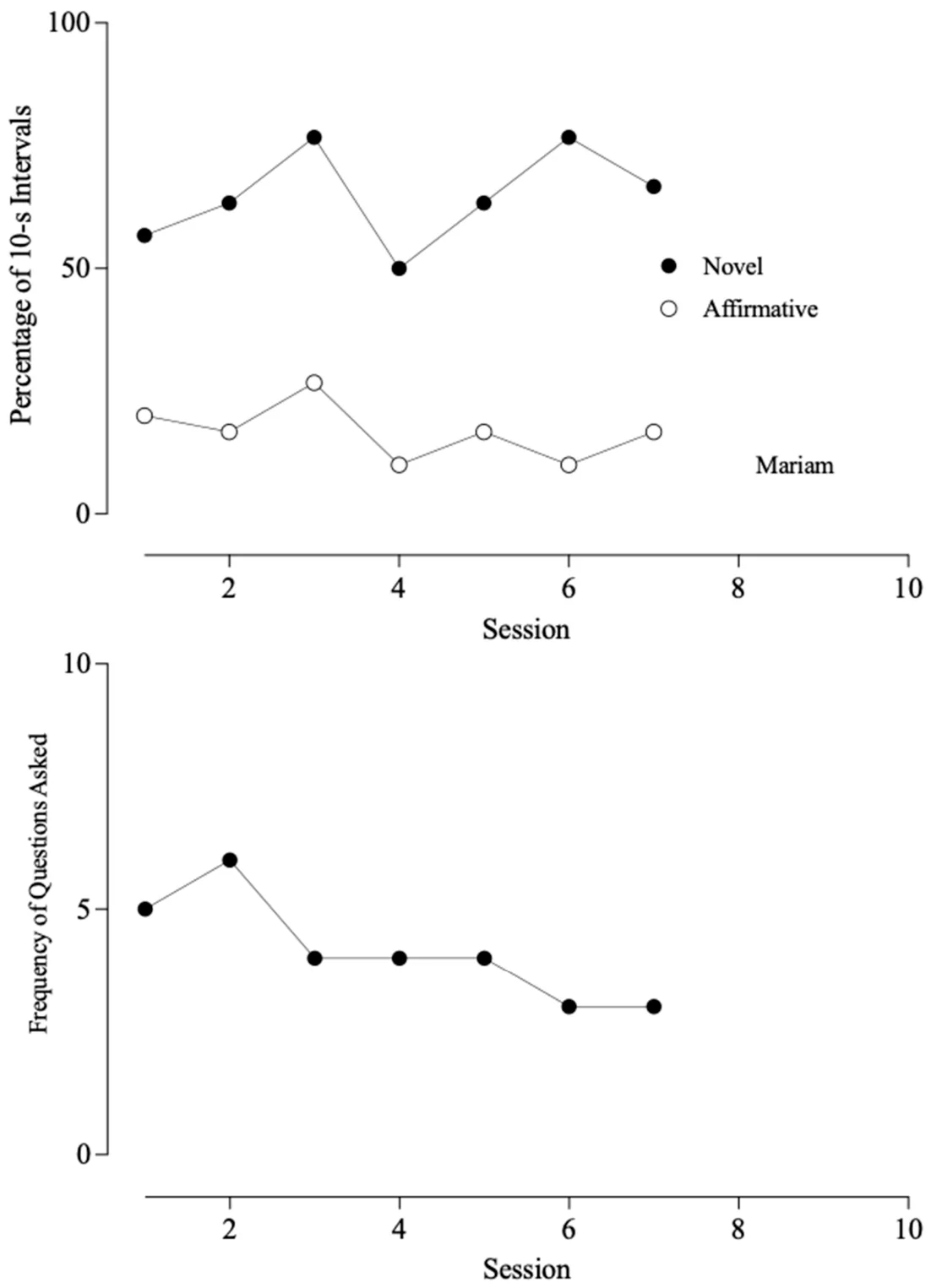
Percent of intervals with Novel and Affirming Vocalizations, and frequency of Questions Asked per session for Mariam.

**Figure 4:**
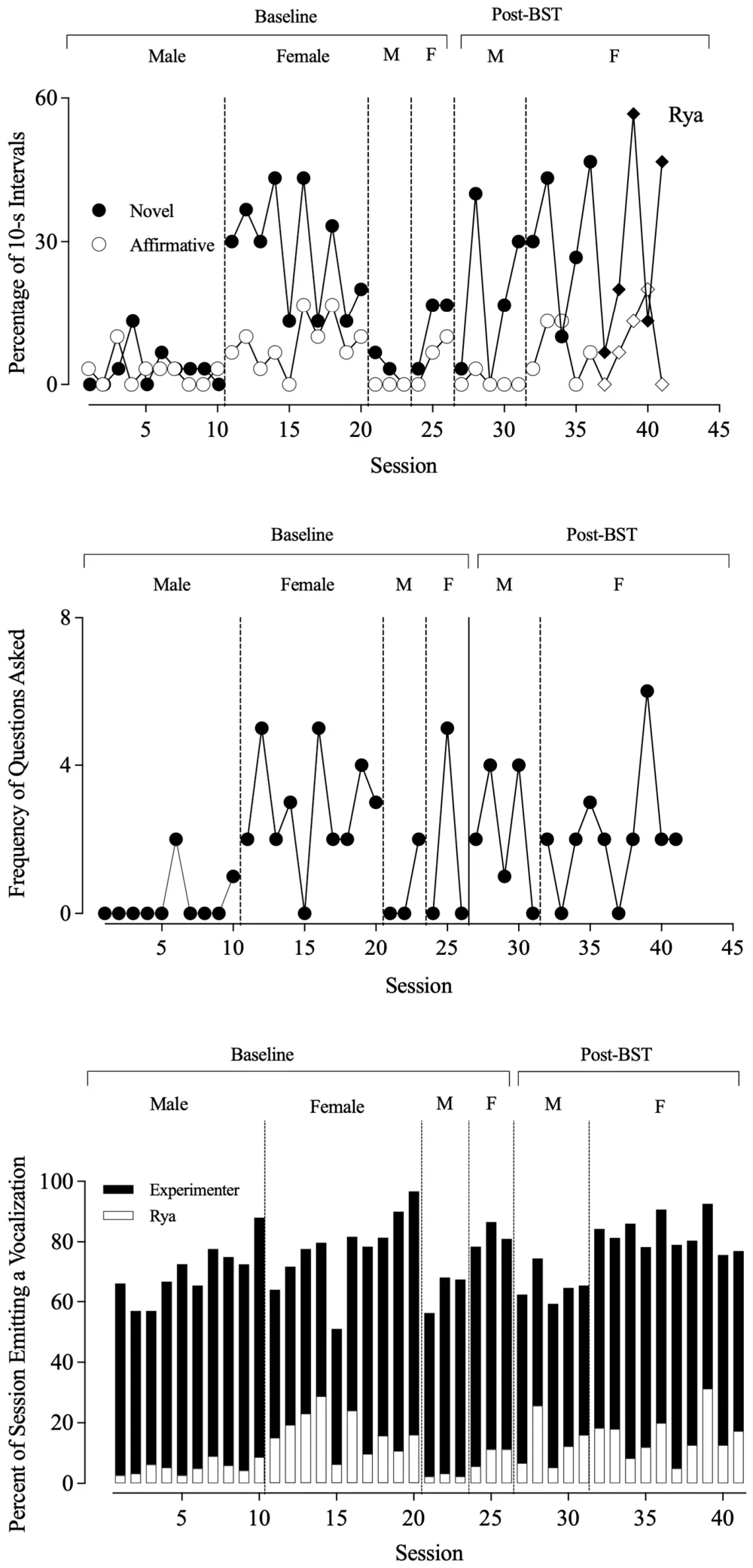
Results for Rya depicting Novel and Affirming Vocalizations and Questions Asked. Bottom panel depicts percent of sessions with participant vocalizations across male- and female-presenting experimenters.

Results for the assessment are depicted in Figure 2, Figure 3 and Figure 4, and Table 1 depicts average novel vocalizations, affirmative vocalizations, and questions asked for all participants. Although formal mastery criteria were not established for the assessment, prior descriptive assessment research (e.g., Hood et al., 2021) may provide useful context for interpreting conversational responding. For example, Hood et al. reported that neurotypical young adults typically asked approximately 0.71–1.44 questions per minute during conversation and maintained high levels of topic-related responding. Thus, increases in reciprocal question asking, sustained novel vocalizations, and greater overall participation in conversation may be interpreted as socially meaningful changes. Assessment outcomes were intended to identify behavioral repertoires that differed substantially from these descriptive benchmarks and therefore may warrant intervention.

Figure 2 depicts results for Jeff. Prior to BST, novel vocalizations occurred during an average of 68% (range, 40–86.7) of intervals per session while affirmative vocalizations occurred during an average of 40% (range, 30–50) of intervals per session. Jeff asked an average of 0.2 (range, 0–1) questions per session. Following BST, we saw a slight increase in novel vocalizations (M = 74.3; range, 60–83.3) and a decrease in affirmative vocalizations (M = 27.7; range, 23.3–33). We also saw a substantial increase in the frequency of questions asked (M = 4; range, 3–5). These findings suggest that Jeff demonstrated strong conversational engagement and novel responding and may have benefited from an intervention targeting reciprocal question asking and conversational maintenance. Following BST, increases in question asking suggested improved participation during conversations.

Figure 3 depicts results for Mariam. Novel vocalizations occurred during an average of 65.8% (range, 50–76.7) of intervals per session and affirmative vocalizations averaged 16.7% (range, 10–26.7) of intervals per session. Mariam asked an average of 4.1 (range, 3–6) questions per session. Mariam chose not to participate in BST, but the assessment showed fairly high levels of all targeted conversational skills. Mariam demonstrated consistent novel vocalizations and reciprocal question asking, suggesting that an intervention to target current conversation components was not necessary. Instead, if intervention was pursued, assessment procedures targeting more advanced social-communication skills may be warranted.

Results for Rya are depicted in Figure 4. Rya’s mother reported that Rya was less confident conversing with male-presenting individuals relative to female-presenting individuals. Therefore, we implemented a reversal design such that Rya conversed with male- and female-presenting individuals. In the presence of male-presenting experimenters during baseline, Rya’s novel vocalizations averaged 3.3% (range, 0–13.7), affirmative vocalizations averaged 2.0% (range, 0–10), and Rya asked an average of 0.38 (range, 0–2) questions per session. In the presence of a female-presenting experimenter, Rya’s novel vocalizations during baseline averaged 24.1% (range, 3.3–43.3), affirmative vocalizations averaged 7.9% (range, 0–16.7), and Rya asked an average of 2.5 (range, 0–5) questions per session. Overall, Rya emitted vocalizations for an average of 4.7% of sessions during baseline with a male-presenting conversation partner and an average of 15.1% of sessions with a female-presenting conversation partner. These findings suggest that Rya’s conversation responding may have been influenced by the characteristics of the conversation partner. The assessment identified potential intervention targets, particularly with male partners, to be reciprocal question asking, conversation engagement, and novel responding.

After BST, in the presence of male-presenting experimenters, Rya’s novel vocalizations averaged 18% (range, 0–40), which is more than a five-fold increase; affirmative vocalizations averaged 0.7% (range, 0–3.3), and she asked an average of 2.2 (range, 0–4) questions per session, which is also more than a five-fold increase. In the presence of a female-presenting experimenter, Rya’s novel vocalizations increased to 30% (range, 6.7–56.7), affirmative vocalizations were 7.7% (range, 0–20), and she asked an average of 2.1 (range, 0–6) questions per session. Overall, Rya emitted vocalizations for an average of 13.2% of sessions with a male-presenting experimenter (more than a two-fold increase) and an average of 15.6% of sessions with a female-presenting experimenter. Following BST, increases in responding were present for novel vocalizations and question asking with male conversation partners. These outcomes demonstrate how the assessment could be used to identify context-specific conversation deficits to help evaluate intervention procedures.

## 4. Discussion

The current study evaluated a brief assessment designed to identify conversational strengths and deficits in adolescents and adults with ASD during small talk interactions. The assessment was sensitive to individual differences in conversational responding across participants and conversation partners. The assessment identified differences in reciprocal question asking, novel vocalizations, and overall conversational engagement, which may help clinicians determine which conversation components require additional support or intervention. Changes observed following BST suggest the assessment may be useful for evaluating clinically relevant changes in conversational reciprocity over time.

Although all dependent variables contributed to understanding conversational responding, novel vocalizations and questions asked appeared to be the most clinically informative measures. Novel vocalizations reflected the participant’s ability to contribute new content to the conversation, whereas questions asked reflected reciprocal engagement with the conversation partner. In contrast, affirmative vocalizations primarily provided information regarding acknowledgment of the partner’s contributions. Across participants, the assessment outcomes and subsequent intervention decisions were most strongly influenced by levels of novel vocal responding and reciprocal question asking.

The clinical utility of this assessment lies in its ability to identify potential deficits in conversational skills that may need to be addressed, which will help to inform more individualized interventions (e.g., BST). The current assessment captured potential deficits in conversational skills related to small-talk conversations across a variety of topics. In practice, clinicians could use assessment outcomes to guide individualized treatment planning by identifying which components of a conversation occur infrequently or require additional prompting. For example, low rates of reciprocal question asking may indicate a need for intervention targeting conversational reciprocity. On the other hand, limited novel vocalizations may suggest a need for an intervention tailored to conversation expansion or maintenance. Most importantly, the assessment provided a necessary framework to evaluate various conversational skills using relatively natural conversation contexts. Future research may evaluate whether this assessment model could be adapted to assess additional conversation behaviors such as prosody (Mann & Karsten, 2021), tacting and responding to sarcasm (Persicke et al., 2013), appropriate body language and facial expressions, and tracking abrupt topic changes, which are all behaviors that are important components of social interactions (Bottema-Beutel, 2020).

The findings highlight the value of individualized assessment procedures when evaluating conversational behavior. Differences in responding across participants and conversation partners suggest that conversational performance may vary as a function of social context and interaction history. Consequently, assessment outcomes may help clinicians tailor intervention goals to the specific conversational needs of each individual rather than relying solely on standardized measures.

However, there are some limitations that future studies will need to address. First, procedural fidelity of the BST sessions was not formally assessed. Although BST was included only as a clinical support procedure rather than as a primary focus of the investigation, the absence of fidelity data limits confidence in the consistency with which BST was implemented across sessions and participants. Future research should include formal treatment-integrity measures to document the extent to which BST procedures are implemented as intended and to better interpret changes observed following training.

Second, although the sessions were designed to approximate natural conversations, sessions still involved a degree of structure, which was necessary to maintain procedural consistency and assessment fidelity. Such structure may have, at times, disrupted the natural flow of conversation. Further, the sessions were limited to 5 min, which allowed for a quick assessment of skills and deficits and a rapid transition to appropriate intervention planning. Although brevity is an important feature of the assessment format (for example, it could be used in a one-hour meeting that is age-appropriate for the individual who participated), the overall time of observation may have produced limited samples of conversational behavior. Nonetheless, the current assessment (or variants of it) would allow clinicians and practitioners to complete the assessment, even with limited resources and time. Based on these limitations, it may be useful to explore ways to make the conversations more natural and less structured, as well as to investigate the effectiveness of different session durations. It may be useful to evaluate the impact of extended sessions to see if more information may be gleaned with additional reciprocal volleys, or, alternatively, if sessions may be shortened to capture similar results in less time.

While we primarily focused on measuring vocal behavior, it is important to acknowledge that communication involves a wide range of non-verbal cues and behaviors that were not analyzed in this study. These non-verbal cues, such as body language, facial expressions, prosody, eye contact, and gestures, play a significant role in conveying meaning, emotions, and intentions in conversations. By omitting the analysis of these non-verbal cues, we may have overlooked valuable information that could further enhance our understanding of effective communication. For instance, body language and facial expressions can provide insights into a person’s engagement, attentiveness, and emotional state during a conversation, while prosody, including intonation, rhythm, and emphasis, can greatly influence the interpretation and emotional impact of spoken words (Sng et al., 2018). Including non-verbal behaviors would be valuable for gaining a comprehensive understanding of communication skills. Future research should consider incorporating the analysis of non-verbal cues and behaviors alongside vocal behavior to provide a more holistic assessment of conversation dynamics. This would enable researchers to capture a broader range of information, leading to a more nuanced understanding of effective communication and facilitating the development of more comprehensive guidelines and interventions for improving conversational skills.

We did not evaluate the cause of the conversation skills deficits. For example, it is unclear whether these deficits were due to a lack of skills or a lack of motivation. Our results suggested that Rya’s reduced vocalizations and question-asking in the presence of male-presenting experimenters indicated she may be experiencing reluctance related to interacting with males. In contrast, her increased vocalizations and question-asking behavior in the presence of female-presenting experimenters suggest that she may feel more comfortable interacting with females. It is important to note that Rya did express to her mother and experimenters that improving her conversational skills would allow her to develop more friendships and possibly a romantic relationship.

The study only employed one type of assessment: reciprocal conversation, specific to engaging in small-talk conversations. Future research could examine the use of this general type of assessment to gain a more nuanced understanding of conversational skills in various social settings, in individuals with (and without, for that matter) ASD. Finally, the current study did not address how to translate assessment results into effective interventions to improve conversational skills in individuals with ASD, as the primary focus was the assessment of conversation skills. We did not focus on BST from the standpoint of experimental design. Further research should explore strategies for developing and implementing such interventions, and for testing their generality across novel contexts, conversational partners, and across time.

Assessing conversational skills in individuals with ASD is crucial for their social and communication development. This assessment is a useful and efficient approach for identifying deficits in conversational skills that may need to be addressed in interventions. The clinical utility of this assessment lies in its ability to provide a naturalistic assessment of conversational skills, identifying potential deficits that may not be captured in more structured assessments or interviews with care providers or participants themselves.

## Figures and Tables

**Table 1:** Average Novel Vocalizations, Affirmative Vocalizations, and Questions Asked for each participant. Novel and Affirmative Vocalizations are depicted as percentages, and Questions Asked are depicted as frequencies. Bold values represent post-BST averages.

Participant	Novel Vocalizations	Affirmative Vocalizations	Questions Asked
Jeff (Pre-BST)	68	40	0.2
Jeff (Post-BST)	**74.3**	**27.7**	**4.0**
Mariam	65.8	16.7	4.1
Rya (Male; Pre-BST)	3.3	2.0	0.38
Rya (Male; Post-BST)	**18.0**	**0.7**	**2.2**
Rya (Female; Pre-BST)	24.1	7.9	2.5
Rya (Female; Post-BST)	**30.0**	**7.7**	**2.1**

## Data Availability

All data generated or analyzed during this study are included in this published article.

## Note

1 Language preferences in the autism community are diverse. As participants in this study varied in their preference for identity-first (“autistic individuals”) versus person-first (“individuals with autism”) terminology, we have retained “individuals with autism” for consistency throughout the manuscript.
